# Phagocytosis of full-length Tau oligomers by Actin-remodeling of activated microglia

**DOI:** 10.1186/s12974-019-1694-y

**Published:** 2020-01-08

**Authors:** Rashmi Das, Abhishek Ankur Balmik, Subashchandrabose Chinnathambi

**Affiliations:** 1 Neurobiology Group, Division of Biochemical Sciences, CSIR-National Chemical Laboratory (CSIR-NCL), Pune, 411008 India; 2 Academy of Scientific and Innovative Research (AcSIR), Pune, 411008 India

**Keywords:** Tau oligomers, Tauopathy, Actin, Microglia, Activation, Migration, Neurodegeneration, Alzheimer’s disease

## Abstract

**Background:**

Alzheimer’s disease is associated with the accumulation of intracellular Tau tangles within neurons and extracellular amyloid-β plaques in the brain parenchyma, which altogether results in synaptic loss and neurodegeneration. Extracellular concentrations of oligomers and aggregated proteins initiate microglial activation and convert their state of synaptic surveillance into a destructive inflammatory state. Although Tau oligomers have fleeting nature, they were shown to mediate neurotoxicity and microglial pro-inflammation. Due to the instability of oligomers, in vitro experiments become challenging, and hence, the stability of the full-length Tau oligomers is a major concern.

**Methods:**

In this study, we have prepared and stabilized hTau40^WT^ oligomers, which were purified by size-exclusion chromatography. The formation of the oligomers was confirmed by western blot, thioflavin-S, 8-anilinonaphthaalene-1-sulfonic acid fluorescence, and circular dichroism spectroscopy, which determine the intermolecular cross-β sheet structure and hydrophobicity. The efficiency of N9 microglial cells to phagocytose hTau40^WT^ oligomer and subsequent microglial activation was studied by immunofluorescence microscopy with apotome. The one-way ANOVA was performed for the statistical analysis of fluorometric assay and microscopic analysis.

**Results:**

Full-length Tau oligomers were detected in heterogeneous globular structures ranging from 5 to 50 nm as observed by high-resolution transmission electron microscopy, which was further characterized by oligomer-specific A11 antibody. Immunocytochemistry studies for oligomer treatment were evidenced with A11^+^ Iba1^high^ microglia, suggesting that the phagocytosis of extracellular Tau oligomers leads to microglial activation. Also, the microglia were observed with remodeled filopodia-like actin structures upon the exposure of oligomers and aggregated Tau.

**Conclusion:**

The peri-membrane polymerization of actin filament and co-localization of Iba1 relate to the microglial movements for phagocytosis. Here, these findings suggest that microglia modified actin cytoskeleton for phagocytosis and rapid clearance of Tau oligomers in Alzheimer’s disease condition.

## Background

Tauopathies are the class of neurodegenerative diseases, which include Alzheimer’s disease (AD), primary age-related tauopathy, fronto-temporal dementia and Parkinsonism linked to chromosome-17 (FTDP-17), progressive supranuclear palsy (PSP), cortico-basal degeneration, and ganglioglioma [1]*.* Tauopathies are characterized by abnormal accumulation of Tau protein in various locations of the brain, leading to progressive neuronal loss, inflammation, and dementia [2]. Tau is a microtubule-associated protein, which mainly functions in the stabilization of neuronal axons, cargo trafficking, and axonal outgrowth under physiological conditions [3]. Full-length Tau (hTau40^WT^) contains two domains—the C-terminal repeat domain, which interacts with microtubules, and the N-terminal projection domain, which maintains the spatial arrangement of microtubules and helps to keep Tau in soluble state. In AD, post-translational modifications (PTMs) such as mutation and truncation of Tau lead to intermolecular interaction followed by the formation of oligomers and subsequently higher-order aggregates [4–8]. The recent findings evidenced that the accumulation of granular Tau oligomers, which was having a size range from 5 to 50 nm, was increased almost four times in the AD brain as compared to the control group [9, 10]. Oligomers can be secreted from neurons via various mechanisms such as passive diffusion and exocytosis [11] as well as with neurotransmitters [12]. Other groups have shown that the propagated Tau oligomers lead to reduced long-term potentiation and increased short-term depression effect on cortical neurons, which has been partially blocked by the administration of oligomer-specific antibody [13, 14]. Oligomers are the unstable species with neurotoxicity and inflammatory activity, which acts as a seed for further aggregation [13, 15, 16]. Mirbaha et al. have evidenced that the small size and confirmation of Tau oligomers (mainly, trimer) are ideal for cellular uptake and propagation [17]. It was shown that the exposure of extracellular oligomers can induce the aggregation of intracellular Tau in HEK293T in vitro cell model [18]. The most effectively endocytosed Tau species were globular in structure and having a high molecular weight (HMW) of > 670 kDa, as observed by size-exclusion chromatography and also immunoreactive to oligomer-specific antibody [19]. Engulfed oligomers get degraded by cellular proteostasis machinery [20] while endocytosed Tau oligomers were found to be located more in lysosomal compartment than Golgi bodies [21].

Microglia are the immune cells in CNS, which functions in constant surveillance of synapses and maintenance of tissue homeostasis. Microglia plays an essential role in early neuronal development as well as adult neuronal regeneration [22]. The unusual presence of synaptic molecules and chemokines is sensed by many membrane receptors on resting microglia, acting as activation signals [23]. In AD, the Tau oligomers are escaped from damaged neurons and spread to synaptically connected neurons and in extracellular space [24]. The extracellular presence of toxic oligomers acts as a conformational template to convert monomeric Tau into the amyloidogenic aggregates. Hence, the phagocytosis of evade oligomers would be most important to prevent the propagation into healthy neuronal circuits [25]. A relatively high concentration of extracellular oligomers can activate microglia, which increases the inflammation, antigen presentation, and phagocytosis of extracellular matrix depositions [26]. But, the improper elimination of damaged neurons by triggered microglia results in synaptic loss and oxidative damage [27]. The prolonged activated microglia have faulty lysosomal machinery, which ultimately causes the release of pro-aggregant protein seed species in interstitial milieu [28]. It has been reported that the internalization and degradation of pathological phosphorylated Tau occur by antibody-mediated and complement-mediated opsonization in murine microglia [29, 30]. In a similar way, primary rat microglia and BV2 microglia have been shown to phagocytose extracellular mutant Tau oligomers upon induced activation with lipopolysaccharides (LPS) [31]. Funk et al. have evidenced the antibody-mediated opsonization of HMW Tau oligomers (> 20 mers) by BV2 microglia, but not neurons, which can be considered as potent immunotherapy in tauopathies [32].

Microglia in resting state maintains the ramified structure with small cell body and long extensions, which samples the parenchymal microenvironment without any net displacement [33]. But in tissue injury or plaque deposition, microglia transforms into the ameboid structure with flat protrusion for migrating longer distances and accumulates at the sites of damage [34]. Microglia migrates along the concentration gradient of chemicals such as-fractalkine [35] and non-soluble aggregates via fan-shaped F-actin-rich lamellipodia and filopodia micro-projections [36]. Microglia forms actin-ring containing podosomes, which interact with vinculin and membrane integrins, termed as podonuts involved in ECM degradation and migration for invasion [37]. The formation of F-actin-rich structural components at the front of polarized microglia generates tensile force and retracts its rear extensions for forward movement [38]. Upon tissue injury or protein aggregation, the microglia become activated [39] and migrated by the upregulation of cytoskeletal network with the localized accumulation of calcium-binding protein-Iba1, which in turn mediates the phagocytosis of protein deposits in neurodegeneration [40–42]. Upon activation, different cytokines determine the expression of matrix metalloproteases, cathepsins, and heparanase for tissue hydrolysis, facilitating chemotactic migration of microglia, which leads to phagocytic clearance of damaged cells, protein deposits, and pathogens [43].

Although oligomers are the most reactive and catastrophic species in terms of Tau-mediated neurodegeneration, these are very unstable intermediate species. The main challenges for studying the oligomers are the stability and homogeneity during the in vitro experiment. Hence, the stabilization of hTau40^WT^ oligomers was the prime most important. In this study, we have stabilized the hTau40^WT^ oligomers using glutaraldehyde and characterized by TEM, western blot, and fluorometric assay. Also, we have identified the potentiality of globular hTau40^WT^ oligomers for the internalization by murine microglia N9 cells [44]. Upon phagocytosis, N9 microglia becomes activated and resulted in downstream upregulation of Ca^2+^-dependent receptor Iba1. Here, we have further investigated the potential of Tau species in actin remodeling, such as the lamellipodia- and filopodia-like structures, which are involved in active migration and phagocytosis of extracellular amyloids in activated N9 microglia.

## Materials and methods

### Chemical and reagents

Luria-Bertani broth (Himedia), ampicillin, NaCl, phenylmethylsulfonylfluoride (PMSF), MgCl2, ammonium persulfate (APS), heparin (17,500 Da), DMSO, and methanol were purchased from MP Biomedicals; IPTG and dithiothreitol (DTT) from Calbiochem; MES, BES, glutaraldehyde, and SDS from Sigma; and EGTA, protease inhibitor cocktail, Tris base, 40% acrylamide, and TEMED from Invitrogen. For cell culture studies, Dulbecco’s modified Eagle’s media (DMEM), fetal bovine serum (FBS), horse serum, phosphate buffer saline (PBS, cell biology grade), trypsin-EDTA, and penicillin-streptomycin were purchased from Invitrogen. Thioflavin-S, ANS, and TritonX-100 were purchased from Sigma. The coverslip of 0.17 mm for immunofluorescence study and copper-coated carbon grids for TEM study were purchased from Blue star and Ted Pella, Inc., respectively. In immunofluorescence and western blot study, we used the following antibodies: total pan-Tau antibody K9JA (Dako, cat. no. A0024), A11 oligomer specific antibody (Thermo, cat. no. AHB0052), Tau antibody (T46) (Thermo, cat. no. 13-6400), Iba1 Polyclonal Antibody (Thermo, cat. no. PA5-27436), β-actin loading control monoclonal antibody (BA3R) (Thermo, cat. no. MA5-15739), Goat anti-rabbit IgG (H + L) cross-adsorbed secondary antibody HRP (Invitrogen, cat. no. A16110), anti-mouse secondary antibody conjugated with Alexa flour-488 (Invitrogen, cat. no. A-11001), Goat anti-rabbit IgG (H + L) cross-adsorbed secondary antibody with Alexa Fluor 555 (Invitrogen, cat. no. A-21428), DAPI (Invitrogen, cat. no. D1306), and ECL reagent (Bio-Rad, cat. no. 1705060). The N9 microglial cell line no. is CVCL_0452.

### Preparation of hTau40^WT^ oligomers and aggregates

The human Tau40^WT^ protein was expressed in *E. coli* BL21* with 100 μg/ml of ampicillin antibiotic selection while the protein expression was induced by 0.5 mM IPTG for 3 h at 37 °C as described earlier [45, 46]. In brief, the bacterial cells were harvested and lysed at 15 kpsi in the Constant cell disruption system (Constant Systems Ltd.). The lysate was heated at 90 °C, centrifuged at 45,000 rpm for 45 min, and dialyzed overnight at 4 °C while the purification of hTau40^WT^ was done by cation-exchange chromatography, followed by size-exclusion chromatography. Tau oligomers were prepared by inducing with polyanionic heparin (17.5 kDa) in phosphate buffer saline (pH 7.4) containing 137 mM NaCl, 3 mM KCl, 10 mM Na_2_HPO_4_, and 2 mM KH_2_PO_4_ added with 2 mM DTT and incubated at 37 °C for 12 h. After the incubation, the hTau40^WT^ oligomers were stabilized using 0.01% glutaraldehyde for 10 min. The solubility and the mass nature of the oligomers were checked for pelleting assay at 60,000 rpm for 1 h, and the sedimented as well as soluble fractions were analyzed by 10% SDS-PAGE. The purified hTau40^WT^oligomers were stored at − 80 °C as 20 μl aliquoted fractions. The hTau40^WT^ preformed aggregates were prepared similarly as oligomerization but in BES buffer (pH − 7.4) by inducing with heparin for 7 days at 37 °C.

### Characterization of oligomers by western blot and fluorometric assay

The hTau40^WT^ oligomers were subjected to size-exclusion chromatography (SEC) (Sephadex 200 Increase, 24 ml) to separate the oligomers from monomers. After SEC, oligomer-containing fractions were subjected to western blot, probed using K9JA total Tau antibody. Only the oligomer-containing fractions were concentrated by 10 kDa MWCO centrifugal filter. The cross-β sheet was formed within the compact fibrillar core of Tau species, which were characterized by thioflavin-S at Ex/Em 440/521 nm. The formation of Tau oligomers is accompanied by the exposure of hydrophobic patches on the surface, which induces the process of multimerization. The hydrophobicity of Tau oligomers and aggregates was observed by ANS fluorescence at Ex/Em 375/490 nm using fluorescence spectrophotometer (Infinite® 200 M PRO, Tecan).

### Conformational analysis of Tau oligomers by circular dichroism spectroscopy

The hTau40^WT^ oligomers, aggregates, and monomers were diluted at a final concentration of 0.15 mg/ml in 50 mM sodium phosphate buffer (pH 6.8) for studying the protein conformational changes. The CD spectral measurements were taken at 20 °C in J-815 spectropolarimeter (JASCO) equipped with Peltier temperature controller. Spectra were measured at a bandwidth of 1 nm with a scan speed of 100 nm/min. The final spectrum depicted the average of 5 acquisitions in the range of 190 to 250 nm [46].

### Tau oligomers and aggregates were mapped by TEM and HR-TEM

The structure of the oligomers was observed by high-resolution transmission electron microscopy where the oligomers and Tau fibrils were spotted onto carbon-coated copper grids (Ted Pella, Inc). An electron-dense layer was provided to Tau oligomers by staining with 2% uranyl acetate. The control samples such as hTau40^WT^ soluble, Tau aggregates induced by heparin and only uranyl acetate were also spotted onto the grids. The grids were dried and analyzed by Tecnai T20 at 200 kV for TEM and JEM-F200 Multi-purpose Electron Microscope (JEOL) for HR-TEM study.

### Tau oligomers quantified by dot blot assay

The hTau40^WT^ oligomers and aggregates were spotted onto a nitrocellulose membrane at a final concentration of 1 mg/ml and allowed for complete drying. The blot was blocked with 5% skimmed milk buffer in 1X PBS with 0.1% Tween 20 for 1 h and probed with A11 (1:1000) and K9JA (1:8000) antibody for 1 h. The blots were further probed with anti-rabbit secondary antibody and developed using ECL reagent (BioRad), and chemiluminescence was recorded by Amersham Imager 600.

### Microglial internalization of hTau40^WT^ oligomers by immunofluorescence

Mice microglial cell line (N9) was passaged in RPMI-1640 media, supplemented with 10% FBS and 100 μg/ml penicillin-streptomycin. For the internalization immunofluorescence experiment, microglia (50,000 cells/well) were seeded onto the coverslip and treated with 1 μM concentration of hTau40^WT^ oligomers and preformed aggregates as a control for 24 h. After incubation, the cells were washed with PBS thrice and fixed with chilled absolute methanol for 10 min. The cells were permeabilized with 0.2% Triton-X 100 and blocked with 2% horse serum. Microglia were immunostained with A11 (1:100) and T46 (1:250) Tau antibody along with nuclear counterstain DAPI. The intracellular localization of A11-specific oligomers was observed by 3D image analysis in Zeiss Axio observer microscope 7 with apotome 2.0. The number of A11^+^ phagocytic cells was counted in multiple fields (*n* = 10), and the percentage of phagocytosis by microglia was represented for hTau40^WT^ oligomer- and aggregate-treated groups. The absolute intensity of A11 and the corresponding area of microglia were quantified by ZEN 2.3 software for the phagocytosis of oligomer.

### Western blot analysis for Iba1 level

N9 cells were treated with hTau40^WT^ oligomers and aggregates at 1 μM concentration for 24 h. The cell lysates for respective treatment groups and control were prepared by adding RIPA cell lysis buffer. The equal amount of protein lysate was loaded in SDS-PAGE and subjected to western blot with Iba1 antibody (1:1000) along with β-actin (1:2500) as a loading control. The relative quantification of Iba1 was done with respect to actin in triplicates, and hence, the graph was plotted.

### Iba1-actin mediated phagocytosis

To study the microglial activation and migration upon hTau40^WT^ exposure, N9 microglial cells were exposed to oligomers and aggregates the same as described above. Then, the microglial actin network was studied for active migration by immunostaining with the β-actin antibody (1:250). The microglial Iba1 was analyzed in the Zeiss Axio observer 7 microscope with apotome 2.0. Similarly, the intensity of actin, Iba1, and associated surface area of 3 microglia per field (*n* = 10 fields) was monitored by ZEN 2.3 software for the identification of migratory growth axis followed by microglial activation. The localization of actin and Iba1 in N9 cells was also observed by immunofluorescence study.

### Statistical analysis

All experiments were done in two biological replicates, and each measurement for every experiment was taken in triplicate. Statistical analyses were performed for fluorometric assay and microscopic quantification by using one-way ANOVA. The statistical significance of the multiple groups has been calculated by Tukey-Kramer’s post hoc analysis for multiple comparisons at 5% level of significance. Each data points (*n* = 8) were plotted in the fluorometric assay. In immunofluorescence microscopic analysis, each data points corresponding to the mean of each field (3 cells/field) and 10 fields were taken into consideration (*n* = 30). The test groups were compared with untreated control, and the *p* values were mentioned within the figures. The results are considered significant if the mean difference between groups is greater than calculated Tukey’s criterion (X − X` > T).

## Results

### Preparation and stabilization of hTau40^WT^ oligomers

**Fig. 1:**
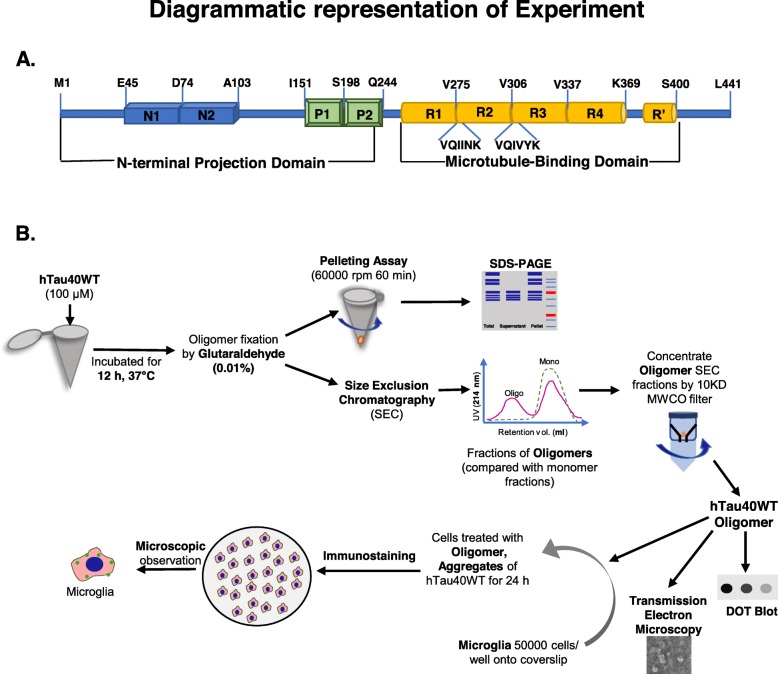
Full-length Tau protein and diagrammatic representation of the experiment. **a** Full-length Tau protein (hTau40^WT^) is a 45-kDa microtubule-binding axonal protein. The hTau40^WT^ has a flexible N- and C-terminal domain organization with which it plays a role in cargo transport and microtubule stabilization, respectively. N-terminal region contains two inserts, and C-terminal includes four repeat regions, which mediate microtubule interaction in physiological condition and also intermolecular interaction leading to oligomerization in AD condition. The two-hexapeptide motif (VQIINK and VQIVYK) in the repeat region plays a critical role in the initiation of Tau oligomerization. **b** Diagrammatic representation illustrates the workflow for oligomer preparation, where hTau40^WT^ was induced with heparin for oligomerization for 12 h. Then, the oligomers were separated by size-exclusion chromatography. Western blot, ThS, and ANS fluorescence were used to characterize the oligomers. The size of the oligomers was confirmed by transmission electron microscopy, and the oligomers were mapped by A11 oligomer-specific antibody

**Fig. 2:**
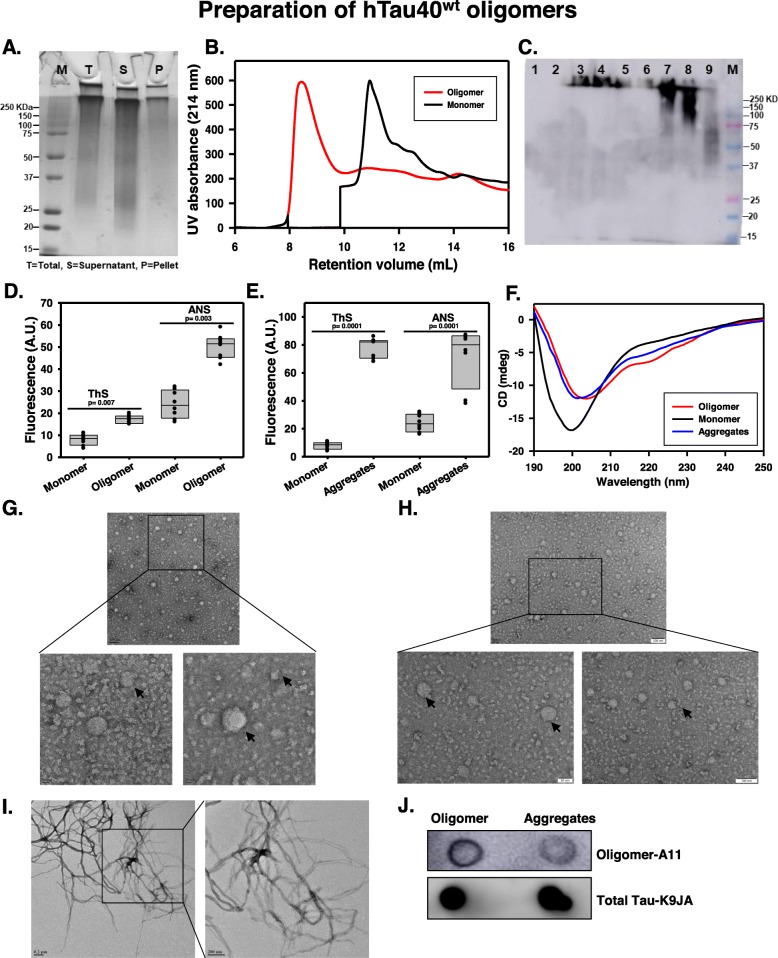
Preparation of full-length Tau oligomers. **a** The oligomerization of hTau40^WT^ was induced by heparin for 12 h, where the high molecular weight Tau oligomers were separated as soluble fractions by ultracentrifugation. **b** Glutaraldehyde-fixed Tau oligomers were separated by size-exclusion chromatography (SEC) with lower elution volume as compared to monomeric fractions. **c** Oligomers containing SEC fractions were confirmed by K9JA western blotting. **d** The formation of internal cross-β structure and the increased hydrophobicity of hTau40^WT^ oligomers were confirmed by fluorometric ThS and ANS assay, respectively. **e** The preformed fibrillar aggregates have shown eightfold and threefold increase in ThS and ANS assay, respectively, as compared to monomers. **f** CD spectroscopic studies revealed the transition from random coil to the β-sheet structure during the process of hTau40^WT^ oligomerization and aggregation as compared with monomeric control. **g**, **h** hTau40^WT^ oligomers formed a globular structure with varying size range 5–50 nm as observed by TEM and HR-TEM. **i** hTau40^WT^ aggregate formed long fibrillar structures, as seen by uranyl acetate negative staining. **j** The hTau40^WT^ oligomers were checked by dot blot staining for the A11 antibody. Significant at the mean difference between treatment groups (X − X`) > Tukey’s criterion (T)

Microtubule-associated protein Tau interacts with tubulin dimers by two hexapeptide motifs localized in repeat 2 and 3 regions at the C-terminal domain in physiological condition. But under pathological conditions, these two motifs play an essential role in the intermolecular oligomerization and further aggregation (Fig. 1a). For Tau aggregation, oligomerization is a rate-limiting step, but once initiated, it accelerates Tau aggregation. Oligomers are very unstable intermediates with a short self-life, formed during the Tau aggregation [47]. Oligomers can be transmitted through synapses in neuronal circuits where they act as a template for endogenous Tau tangle formation and released into extracellular space [24]. Tau oligomers have found to be increased in age-dependent AD brain, which resulted in NFT formation and subsequent neurodegeneration [13, 48]. The P301S Tau mice model showed the hippocampal synaptic loss, axonal spheroid formation, and activated microglial phenotype before the onset of Tau tangle formation in the brain [49]. The Tau oligomers which are isolated from AD brain have induced cytotoxicity in neuroblastoma cell line at a lower concentration, which has been reversed by oligomer-specific antibody TOMA [50]. Hence, the trapping of reactive oligomers and their subsequent degradation by tissue-resident microglia would be of great importance to prevent the AD pathology. To form the stable oligomers, hTau40^WT^ monomers in reducing condition were induced for oligomerization with polyanionic co-factor heparin in PBS (pH 7.4) [51] as represented by diagrammatic workflow (Fig. 1b). The formation of soluble hTau40^WT^ oligomer was evidenced after 12 h of incubation as depicted by pelleting assay (Fig. 2a). The hTau40^WT^ oligomers were stabilized by 0.01% glutaraldehyde for in vitro experiments. When the oligomer reaction mixture was allowed to pass through SEC, it was eluted out at lower retention volume of 8.2 ml as compared to the corresponding monomer fractions at 12.1 ml retention volume. A distinct peak shift was evidenced in the SEC, which indicates the formation of higher-order oligomers (Fig. 2b). The formation of oligomers was characterized by WB analysis of SEC fractions with a total pan-Tau K9JA antibody (Fig. 2c).

### hTau40^WT^ oligomers evidenced by fluorometric, CD spectroscopy, TEM, and DOT blot assay

Thioflavin-S (ThS) is a fluorophore, which specifically binds to the compact cross-β structure formed inside the core of the misfolded Tau aggregates while the cross β-structures are transient in oligomers [52]. Therefore, thioflavin is used for fluorometric detection of amyloidogenic aggregation kinetics in vitro as well as the localization of amyloid deposits in AD brain [53]. As the oligomers are very fragile species, the ThS fluorescence did not significantly increase with oligomers as compared to the hTau40^WT^ monomeric control (ThS to protein = 4:1 M ratio). But, the ANS fluorescence, which is an indicator of exposed surface hydrophobicity on protein aggregates [54], oligomers showed increased ANS fluorescence by twofold with respect to monomers in same concentration (ANS to protein = 20:1 M ratio). Collectively, fluorometric assay depicted the formation of hydrophobic oligomers with a lesser extent of β-sheet structures (Fig. 2d). Similarly, the mature Tau fibrils also have the high content of internal compact β-sheet structures and hydrophobicity, detected by ThS and ANS fluorometric assay, respectively (Fig. 2e). The oligomerization of amyloidogenic protein-Tau induces the formation of β-sheet structures, which is an indication of the kinetic acceleration of converging toxic intermediates in AD. CD spectroscopy helps to understand the overall molecular conformations of proteins, notably the transition from random coil to β-sheet structures in the case of Tau oligomers [48]. Tau monomers showed minimum ellipticity at 200 nm as seen in the spectral curve, which is an indication of random coil structures, while hTau40^WT^ oligomers and aggregates showed the minimum ellipticity at 204 nm and a growing “shoulder” at 220 nm, which indicates the tendency towards β-sheet formation during oligomerization (Fig. 2f).

As the truncated Tau [31] and repeat Tau domains [51] have been already reported to form globular oligomeric structures [55], we were interested in identifying the nanostructures of hTau40^WT^ oligomers. TEM (Fig. 2g) and HR-TEM (Fig. 2h) analysis revealed that the hTau40^WT^ oligomers have formed heterogeneous globular structures ranging from 5 to 50 nm (Additional file 1: Fig. S1A) while the elongated fibrils (Fig. 2i) were absent in oligomer preparation. The soluble Tau (Additional file 1: Fig. S1B) and only uranyl acetate staining (Additional file 1: Fig. S1C) control did not show a specified structure by TEM study. The oligomers were characterized by oligomer-specific A11 antibody in dot blot analysis [54], where hTau40^WT^ oligomers were more stained with A11 antibody as compared to preformed aggregates when probed at the same concentration of 1 mg/ml as compared to the total-Tau K9JA control staining (Fig. 2j).

### Internalization of hTau40^WT^ in microglia

**Fig. 3:**
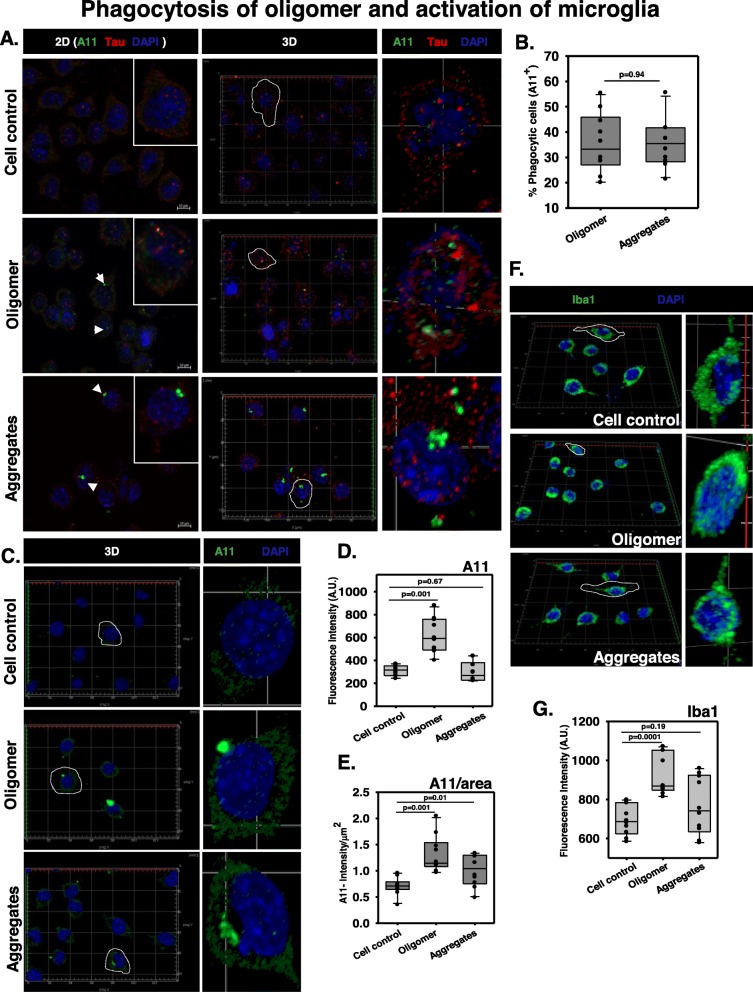
Phagocytosis of Tau oligomers and activation of microglia. **a** N9 microglia were incubated with extracellular Tau oligomers for 24 h to initiate phagocytosis. The immunostaining study analyzed the presence of A11^+^ microglia indicating the phenomenon of active internalization of oligomer overburden. **b** The number of A11+ microglia was equal in amount during the phagocytosis of hTau40^WT^ oligomers and fibrillar aggregates. **c** 3D localization studies have shown that the microglia have engulfed an adequate amount of Tau oligomers and low molecular weight aggregates (A11 staining) as compared to the untreated control group. **d**, **e** The quantification of total A11 intensity in microglia upon different Tau species treatment group and the A11 intensity per square area have defined the density of A11 fluorescence in treated microglia by ZEN 2.3 image analytical microscopic software. **f**, **g** Microglial activation is mediated by the peri-membrane localization of Ca^2+^ binding adaptor protein Iba1 in the case of preformed Tau aggregate treatment while the oligomer-treated group showed an increased level of cytosolic localization of Iba1 in microglia. The levels of Iba1 were quantified by ZEN 2.3 image analysis software in N9 microglia treated with Tau species. Significant at the mean difference between treatment groups (X − X`) > Tukey’s criterion (T)

Microglia are the CNS-resident macrophages, which function to phagocytose unusual matrix deposition, invading pathogens, and improper synapses, as well as the damaged and degenerating neurons [56]. The secretion of the intracellular soluble Tau oligomers can be considered as an early event of tauopathy [57], which can act as an activation signal for microglia. Microglia mainly functions to degrade the engulfed protein clumps by the lysosomal pathway and eventually results in the activation of inflammation with rapid clearance [58]. Recent evidence proved that the Tau species can be internalized by dynamin-mediated endocytosis, which is independent of actin polymerization-mediated micropinocytosis [59]. It can also be internalized through interacting with 6-O-heparan sulfate proteoglycan followed by the involvement of lipid microdomain [60]. To study the internalization of hTau40^WT^ oligomers by N9 cells, microglial cells were incubated with hTau40^WT^ oligomers or aggregates for 24 h and the intracellular localization of A11^+^ oligomers were studied by IF staining. Immunocytochemistry study depicted the intracellular 3D localization and surface adherence of A11^+^ oligomers in/on microglia as compared to the untreated group (Fig. 3a). The 38% of microglia were found to be A11^+^ in oligomer-treated group while the fibrillar aggregate treatment has shown the phagocytosis by 35% of microglia (Fig. 3b). Due to the presence of heterogeneous species in preformed aggregates, i.e., containing both oligomers and mature fibrils, we have also observed A11^+^ microglia in aggregate-treated group (Fig. 3c). The A11 intensity was increased in microglia by oligomer- and aggregate-treated groups in contrast to the untreated group (Fig. 3d, e). These results cumulatively suggest that the microglia could exhibit the phagocytic engulfment of extracellular hTau40^WT^ oligomers for clearance.

### Microglial activation by engulfed Tau oligomers

Extracellular Tau oligomers can act as an activation signal for microglia. Microglial activation leads to the orchestration of various membrane receptors as well as the secretion of different cytokines and chemokines, attracting neighboring microglia to speed up the process of the active clearance of deposited misfolded proteins and degenerated neuronal bodies [61]. Intracellular calcium level plays an important role in microglial migration activation [62], and a calcium-dependent adaptor protein, Iba1, was found to be upregulated by the overexpression of hTau40 in microglia [63]. It was reported that the IFN-γ, IL-1β, and tissue injury could increase the Iba1 levels related to cellular proliferation and migration [64]. In our study, we found that the Tau aggregate treatment has spread the Iba1 localization towards the cell membrane projection (Fig. 3f), while the oligomer-treated group showed increased cytosolic levels of Iba1 in microglia compared to resting state (Fig. 3g). This observation suggests that Tau pathological species can drive the activation of microglia during AD condition.

### Microglia phagocytosed hTau40^WT^ oligomers by modulating actin network

**Fig. 4:**
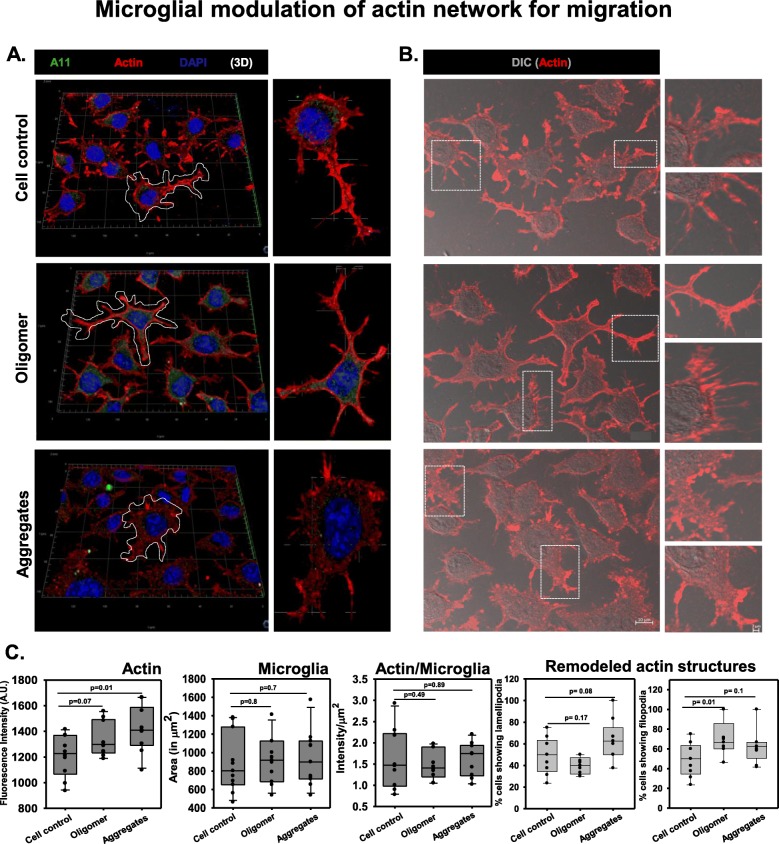
Microglial modulation of actin network for engulfment and migration. **a**, **b** Microglia, upon Tau aggregate treatment, formed increased fan-shaped lamellipodia-like actin structure at the site of growth and movement while oligomer-treated group has shown with only micro-spike actin extensions called filopodia, which may signify the initiation process of phagocytosis. **c** Microscopic quantification depicted the increase of actin intensity in microglia upon oligomer and aggregate exposure, but when normalized with a cellular surface area, the actin levels were non-significantly altered. **d** These results signified only the remodeling of the actin network in lamellipodia-like and podosome-like migratory structures for phagocytic clearance of Tau overburden. Significant at the mean difference between treatment groups (X − X`) > Tukey’s criterion (T)

**Fig. 5:**
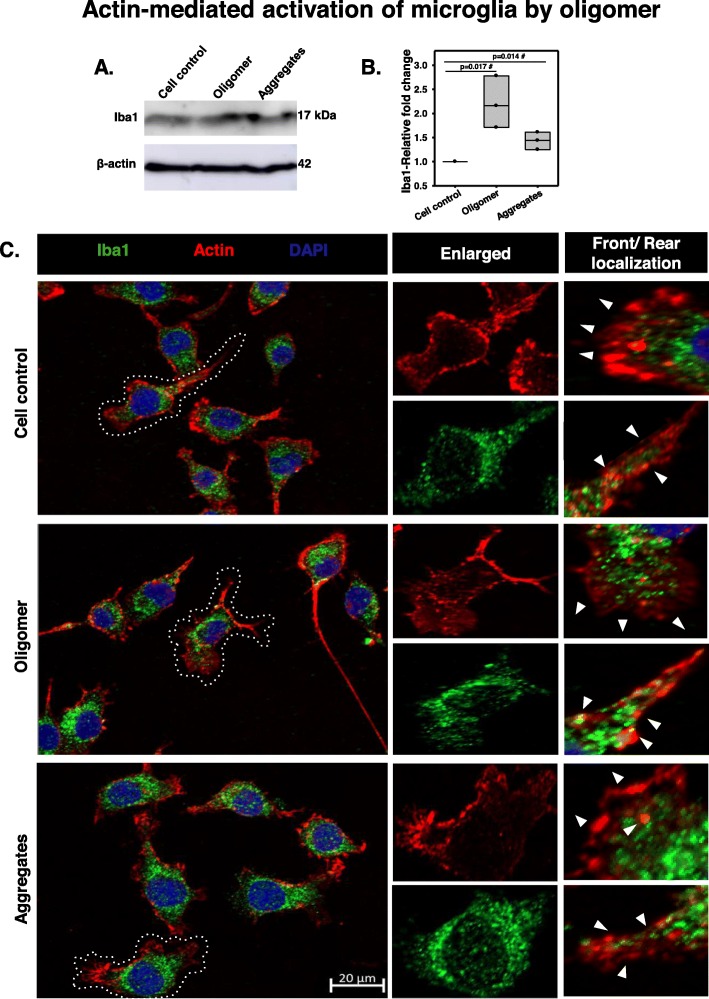
Actin-mediated activation of microglia by Tau oligomers. **a**, **b** N9 microglia showed an increased level of Iba1 in hTau40^WT^ oligomer-treated group as compared to aggregate exposure by western blot. Significant at the mean difference between treatment groups (X − X`) > Tukey’s criterion (T). The # sign corresponds to non-significant by Tukey’s test. **c** The immunostaining study revealed the accumulation of Iba1 with remodeled actin structures at the rear end of activated migratory microglia. The localization of Iba1 at the rear site of microglia may emphasize the contraction of the actin network for mediating forward movements during migration. The “arrow” has represented the migratory direction in front ends and the retraction of actin structures containing Iba1 from rear ends in N9 cells

Actin is one of the basic cytoskeletal network involved in the cellular structure maintenance and direction determination for the cell motility. The actin remodeling may be beneficial for microglial migration, matrix adhesion, and phagocytic clearance of extracellular protein burdens [65]. It was reported that the repopulation of senescent microglia improves the age-related neuro-synaptic cytoskeleton remodeling and dendritic spine regeneration without altering immune activation and phagocytosis in glia [66, 67]. In order to check whether oligomeric species can exhibit the migratory state of N9 microglia [68, 69], the actin network was stained for hTau40^WT^ oligomer- and aggregate-treated groups. Here, we observed that Tau aggregate treatment resulted in microglial membrane extension with increased fan-shaped actin-rich lamellipodia-like structure, which might suggest the microglial migration for phagocytosis of extracellular Tau species (Fig. 4a). The oligomer-treated group has shown to increase actin micro-spikes, which can be termed as filopodia-like structure (Fig. 4b), which signifies the initiation of microglial motility. Treatment with Tau species apparently resulted in increased actin intensity in N9 microglia. However, the actin levels normalized to the surface area remained unchanged, signifying only the rearrangement of the actin network in podosome-like structure for active phagocytosis of extracellular Tau oligomers and fibrils by migration (Fig. 4c, d). In the western blot study, the levels of Iba1 in N9 cells were upregulated in oligomer-treated group as compared to aggregate exposure for 24 h. The relative quantification, followed by statistical significance analysis, clarified that the altered level of Iba1 was insignificant among all treatment groups (Fig. 5a, b). Interestingly, immunofluorescence study revealed that Iba1 were found to co-localize more in the rear ends of N9 cells where the cellular extensions were contracted for forward movement. The front actin-containing lamellipodia structures, helping in the forward-direction movement, were observed to contain less co-localization with Iba1 in all treatment groups (Fig. 5c).

## Discussion

Tau interacts with tubulin through C-terminal repeat domain and maintains microtubule stability. But in diseased condition, destabilization of Tau results in the formation of soluble oligomers, followed by insoluble aggregates in synaptic puncta [70]. The reactive nature of oligomers allows interaction and malfunctioning of many cellular proteins, which ultimately leads to release from the damaged neurons and imparting toxic seeding to the neighboring neurons [13]. Additionally, the cellular kinases and cysteine-proteases play a pivotal role in Tau hyperphosphorylation and cleavage, respectively, which allow the protein to become more aggregation-prone and resistant for degradation [20]. Truncated Tau (aa 151–391) has been shown to form globular oligomers in vitro, which are being phagocytosed by LPS-stimulated activated microglia [31]. Similarly, RDΔK280 Tau oligomers were found to attain distinct granular oligomers without β-sheet confirmation, which were responsible for ROS production and synapto-toxicity but not the viability in hippocampal neurons [51]. Due to the flexibility of the full-length Tau domain, hTau40^WT^ oligomers are transient in nature [71]. A 0.01% concentration of glutaraldehyde stabilized oligomers formed in the course of aggregation and purified by SEC [51]. The purified hTau40^WT^ oligomers were confirmed by western blot, ThS, and ANS fluorescence studies. While the high-resolution electron microscopic analysis revealed the presence of heterogeneous oligomers, which are similar kind with small globular hTau40^WT^ oligomers [9], the assembly of β-sheet structures was confirmed by CD spectroscopy [72]. Due to the small size of oligomers, they can be engulfed by microglia, leading to activation, which mimics the pathological status of AD condition [73].

Microglia play a crucial role in neurodevelopment as well as neuro-regeneration after acute tissue insult by secretion of various growth factors. Microglia have a small cell body with the long extensions by which they sense the microenvironment and synaptic transmitter [74]. Upon inflammation or extracellular protein deposition, microglia becomes activated and secretes a series of cytokines and chemokines. Various membrane receptors and complement factors are also orchestrated for the recognition of death-associated signals and amplify the inflammatory burst to initiate improper phagocytosis of synapses [30, 75]. Activated microglia engulf patho-proteins containing neurons flagging phosphatidylserine signals [76] and degrade via lysosomal machinery [58]. But, the failure of proper proteolysis can lead to the excretion of cleaved peptides, which acts as seed entities for further aggregation within healthy neurons [77]. In our study, we found the phagocytosis of extracellular Tau oligomers and adherence of high molecular weight Tau aggregates onto N9 microglial cells, indicating the initiation of active internalization and clearance. Microglia can engulf protein aggregates containing neuronal synapse upon activation, which signals can be taken into consideration for the selection of immune targets as therapeutics [78].

Iba1 is a calcium-binding protein receptor, which functions as a marker for microglia or brain-infiltrating macrophages in the synaptic surveillance [79, 80]. During neuronal injury-mediated inflammation, Iba1 level becomes upregulated in the brain microenvironment, which correlates with the accumulation of migratory microglia and activated serotype into the site of injury [41, 63]. On the contrary, we evidenced the increased level and localization of Iba1 proteins towards the microglial actin-containing membrane projection upon the exposure of extracellular protein aggregates, which may suggest that the Ca^2+^-mediated activation and actin-mediated migration of microglia lead to the rapid clearance of deposits [81].

**Fig. 6:**
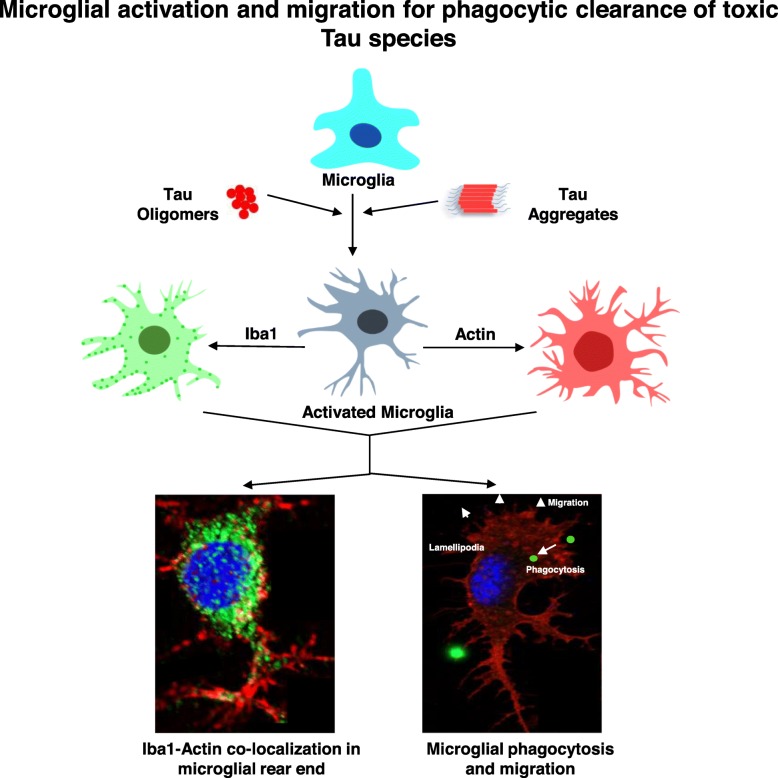
Microglial migration and activation for the phagocytosis of hTau40^WT^ oligomers. Extracellular Tau aggregates or oligomers have induced the activation state of microglia with high Iba1 and modified actin cytoskeleton network, which ultimately leads to the rapid phagocytosis and clearance of Tau oligomers. Extracellular leakage of Tau species from damaged neurons can act as a trigger for microglial activation and migration, which ultimately will be beneficial as a biomarker for early onset and as a target for therapy

Actin is a dynamic cellular cytoskeletal network, which functions to mediate the active migration of the cells. In the migratory growth end of the cells, globular actin (G-actin) rapidly polymerizes into fibrillar actin (F-actin), which makes a tensile strength for the active movement in a particular direction [82], while F-actin depolymerizes into G-actin to increase the membrane fluidity for rapid phagocytosis of extracellular particles [43, 83]. Reorganization of actin filaments provides the emphasis on microglial effector function while several adaptor proteins such as CD36 [84], ROCK [83], and cofilin, interacting with actin podosomes [85], can lead to the events of migration and phagocytosis. Similarly, Iba1 was reported to remodel actin bundling by directly interacting with actin-binding protein l-fimbrin and subsequently induce phagocytic cup formation [81, 86]. Here, we identified the formation of more filopodia-like actin structure and co-localization with Iba1 upon oligomer exposure in N9 cells. But the microglia become more migratory by remodeling actin network with increased fan-like lamellipodia structure for the rapid clearance of preformed Tau fibrillar aggregates (Fig. 6). The increased migration of microglia and Iba1-driven rearrangements of actin networks can provide an insight on microglial functions of phagocytic clearance of amyloidogenic protein species in tauopathy. The extracellular Tau species can act as a signal to brain-resident or brain-infiltrated microglia for enhanced proteostasis and mediate innate-adaptive immune response. The molecular mechanisms of phagocytosis and improvement of microglial motility will also help to identify the possible immune targets in the scenario of tauopathy.

## Conclusion

Considering these results, microglia can phagocytose hTau40^WT^ oligomers effectively, which subsequently altered the increased level of Iba1. The activation of microglia might increase Ca2+-mediated migration through actin podosome-like structure resulting in phagocytic clearance of extracellular Tau overloads.

## Supplementary information

**Additional file 1: Figure S1.** TEM analysis of hTau40^WT^species and control groups. (A) hTau40^WT^ oligomer forms globular structure as observed by TEM. (B) Soluble Tau fixed with glutaraldehyde were negatively stained with uranyl acetate, which formed no specified structures in TEM study. (C) The carbon mess grids stained with only uranyl acetate as negative control. (D) The SDS-PAGE analysis of hTau40^WT^ fibrillar aggregate and soluble monomer

## Data Availability

All data generated or analyzed during this study are included in this manuscript and its Additional file.

## Abbreviations

AD: Alzheimer’s disease
ANS: 8-Anilinonaphthaalene-1-sulfonic acid
Ca^2+^: Calcium ion
CD spectroscopy: Circular dichroism spectroscopy
CNS: Central nervous system
HTau40^WT^: Full-length Tau
Iba1: Ionized calcium-binding adaptor protein1
LPS: Lipopolysaccharides
PBS: Phosphate buffer saline
PTMs: Post-translational modifications
SEC: Size-exclusion chromatography
TEM: Transmission electron microscopy
ThS: Thioflavin-S
WB: Western blot

## References

[1] Hernandez F, Avila J (2007). Tauopathies. Cell Mol Life Sci.

[2] Orr ME, Sullivan AC, Frost B (2017). A brief overview of tauopathy: causes, consequences, and therapeutic strategies. Trends Pharmacol Sci.

[3] Mandelkow E-M, Mandelkow E (2012). Biochemistry and cell biology of tau protein in neurofibrillary degeneration. Cold Spring Harb Perspect Med.

[4] Cohen TJ, Guo JL, Hurtado DE, Kwong LK, Mills IP, Trojanowski JQ, Lee VMY (2011). The acetylation of tau inhibits its function and promotes pathological tau aggregation. Nat Commun.

[5] Rane JS, Kumari A, Panda D (2019). An acetylation mimicking mutation, K274Q, in tau imparts neurotoxicity by enhancing tau aggregation and inhibiting tubulin polymerization. Biochem J.

[6] de Calignon A, Fox LM, Pitstick R, Carlson GA, Bacskai BJ, Spires-Jones TL, Hyman BT (2010). Caspase activation precedes and leads to tangles. Nature.

[7] Hanger Diane P., Wray Selina (2010). Tau cleavage and tau aggregation in neurodegenerative disease. Biochemical Society Transactions.

[8] Combs B, Gamblin TC (2012). FTDP-17 tau mutations induce distinct effects on aggregation and microtubule interactions. Biochemistry.

[9] Maeda S, Sahara N, Saito Y, Murayama S, Ikai A, Takashima A (2006). Increased levels of granular tau oligomers: an early sign of brain aging and Alzheimer’s disease. Neurosci Res.

[10] Lasagna-Reeves CA, Castillo-Carranza DL, Sengupta U, Sarmiento J, Troncoso J, Jackson GR, Kayed R (2012). Identification of oligomers at early stages of tau aggregation in Alzheimer’s disease. FASEB J.

[11] Xia Y, Zhang G, Han C, Ma K, Guo X, Wan F, Kou L, Yin S, Liu L, Huang J (2019). Microglia as modulators of exosomal alpha-synuclein transmission. Cell Death Dis.

[12] Guo JL, Lee VMY (2014). Cell-to-cell transmission of pathogenic proteins in neurodegenerative diseases. Nat Med.

[13] Lasagna-Reeves CA, Castillo-Carranza DL, Sengupta U, Guerrero-Munoz MJ, Kiritoshi T, Neugebauer V, Jackson GR, Kayed R (2012). Alzheimer brain-derived tau oligomers propagate pathology from endogenous tau. Sci Rep.

[14] Hill Emily, Karikari Thomas K., Moffat Kevin G., Richardson Magnus J. E., Wall Mark J. (2019). Introduction of Tau Oligomers into Cortical Neurons Alters Action Potential Dynamics and Disrupts Synaptic Transmission and Plasticity. eneuro.

[15] Michelucci A, Heurtaux T, Grandbarbe L, Morga E, Heuschling P (2009). Characterization of the microglial phenotype under specific pro-inflammatory and anti-inflammatory conditions: effects of oligomeric and fibrillar amyloid-β. J Neuroimmunol.

[16] Jiang L, Ash PE, Maziuk BF, Ballance HI, Boudeau S, Al Abdullatif A, Orlando M, Petrucelli L, Ikezu T, Wolozin B (2019). TIA1 regulates the generation and response to toxic tau oligomers. Acta Neuropathol.

[17] Mirbaha H, Chen D, Morazova OA, Ruff KM, Sharma AM, Liu X, Goodarzi M, Pappu RV, Colby DW, Mirzaei H (2018). Inert and seed-competent tau monomers suggest structural origins of aggregation. Elife.

[18] Mirbaha H, Holmes BB, Sanders DW, Bieschke J, Diamond MI (2015). Tau trimers are the minimal propagation unit spontaneously internalized to seed intracellular aggregation. J Biol Chem.

[19] Takeda S, Wegmann S, Cho H, DeVos SL, Commins C, Roe AD, Nicholls SB, Carlson GA, Pitstick R, Nobuhara CK (2015). Neuronal uptake and propagation of a rare phosphorylated high-molecular-weight tau derived from Alzheimer’s disease brain. Nat Commun.

[20] Sonawane SK, Chinnathambi S (2018). Prion-like propagation of post-translationally modified tau in Alzheimer’s disease: a hypothesis. J Mol Neurosci.

[21] Nobuhara CK, DeVos SL, Commins C, Wegmann S, Moore BD, Roe AD, Costantino I, Frosch MP, Pitstick R, Carlson GA (2017). Tau antibody targeting pathological species blocks neuronal uptake and interneuron propagation of tau in vitro. Am J Pathol.

[22] Jin X, Yamashita T (2016). Microglia in central nervous system repair after injury. The Journal of Biochemistry.

[23] Ben Haim L, Carrillo-de Sauvage M-A, Ceyzériat K, Escartin C (2015). Elusive roles for reactive astrocytes in neurodegenerative diseases. Front Cell Neurosci.

[24] Chen X-Q, Mobley WC. Alzheimer disease pathogenesis: insights from molecular and cellular biology studies of oligomeric Aβ and tau species. Front Neurosci. 2019;13. 10.3389/fnins.2019.00659 PMC6598402 31293377

[25] Holmes BB, Diamond MI (2014). Prion-like properties of tau protein: the importance of extracellular tau as a therapeutic target. J Biol Chem.

[26] Das R, Chinnathambi S. Microglial priming of antigen presentation and adaptive stimulation in Alzheimer’s disease. Cell Mol Life Sci. 2019:1–14. 10.1007/s00018-019-03132-2 PMC11105582 31093687

[27] Leyns CEG, Holtzman DM (2017). Glial contributions to neurodegeneration in tauopathies. Mol Neurodegener.

[28] Chung H, Brazil MI, Soe TT, Maxfield FR (1999). Uptake, degradation, and release of fibrillar and soluble forms of Alzheimer’s amyloid β-peptide by microglial cells. J Biol Chem.

[29] Luo W, Liu W, Hu X, Hanna M, Caravaca A, Paul SM (2015). Microglial internalization and degradation of pathological tau is enhanced by an anti-tau monoclonal antibody. Sci Rep.

[30] Dejanovic B, Huntley MA, De Mazière A, Meilandt WJ, Wu T, Srinivasan K, Jiang Z, Gandham V, Friedman BA, Ngu H (2018). Changes in the synaptic proteome in tauopathy and rescue of tau-induced synapse loss by C1q antibodies. Neuron.

[31] Majerova P, Zilkova M, Kazmerova Z, Kovac A, Paholikova K, Kovacech B, Zilka N, Novak M (2014). Microglia display modest phagocytic capacity for extracellular tau oligomers. J Neuroinflammation.

[32] Funk KE, Mirbaha H, Jiang H, Holtzman DM, Diamond MI (2015). Distinct therapeutic mechanisms of tau antibodies promoting microglial clearance versus blocking neuronal uptake. J Biol Chem.

[33] Prinz M, Mildner A (2011). Microglia in the CNS: immigrants from another world. Glia.

[34] Kettenmann H, Hanisch U-K, Noda M, Verkhratsky A (2011). Physiology of microglia. Physiol Rev.

[35] Xie W-L, Shi Q, Zhang J, Zhang B-Y, Gong H-S, Guo Y, Wang S-B, Xu Y, Wang K, Chen C (2013). Abnormal activation of microglia accompanied with disrupted CX3CR1/CX3CL1 pathway in the brains of the hamsters infected with scrapie agent 263K. J Mol Neurosci.

[36] Chhabra ES, Higgs HN (2007). The many faces of actin: matching assembly factors with cellular structures. Nat Cell Biol.

[37] David-Pfeuty T, Singer SJ (1980). Altered distributions of the cytoskeletal proteins vinculin and alpha-actinin in cultured fibroblasts transformed by Rous sarcoma virus. Proc Natl Acad Sci.

[38] Lauffenburger DA, Horwitz AF (1996). Cell.

[39] Maezawa I, Zimin PI, Wulff H, Jin L-W (2011). Amyloid-β protein oligomer at low nanomolar concentrations activates microglia and induces microglial neurotoxicity. J Biol Chem.

[40] Abd-El-Basset EM, Prashanth J, Lakshmi KVVA (2004). Up-regulation of cytoskeletal proteins in activated microglia. Med Princ Pract.

[41] Seminotti B, Zanatta Â, Ribeiro RT, da Rosa MS, Wyse AT, Leipnitz G, Wajner M (2019). Disruption of brain redox homeostasis, microglia activation and neuronal damage induced by Intracerebroventricular administration of S-Adenosylmethionine to developing rats. Mol Neurobiol.

[42] Ito D, Tanaka K, Suzuki S, Dembo T, Fukuuchi Y (2001). Enhanced expression of Iba1, ionized calcium-binding adapter molecule 1, after transient focal cerebral ischemia in rat brain. Stroke.

[43] Lively S, Schlichter LC (2013). The microglial activation state regulates migration and roles of matrix-dissolving enzymes for invasion. J Neuroinflammation.

[44] Stansley B, Post J, Hensley K (2012). A comparative review of cell culture systems for the study of microglial biology in Alzheimer’s disease. J Neuroinflammation.

[45] Sonawane SK, Ahmad A, Chinnathambi S (2019). Protein-capped metal nanoparticles inhibit tau aggregation in Alzheimer’s disease. ACS omega.

[46] Gorantla Nalini Vijay, Landge Vinod G., Nagaraju Pramod Gudigenahally, Priyadarshini CG Poornima, Balaraman Ekambaram, Chinnathambi Subashchandrabose (2019). Molecular Cobalt(II) Complexes for Tau Polymerization in Alzheimer’s Disease. ACS Omega.

[47] Tepper K, Biernat J, Kumar S, Wegmann S, Timm T, Hübschmann S, Redecke L, Mandelkow E-M, Müller DJ, Mandelkow E (2014). Oligomer formation of tau protein hyperphosphorylated in cells. J Biol Chem.

[48] Maeda S, Sahara N, Saito Y, Murayama M, Yoshiike Y, Kim H, Miyasaka T, Murayama S, Ikai A, Takashima A (2007). Granular tau oligomers as intermediates of tau filaments. Biochemistry.

[49] Yoshiyama Y, Higuchi M, Zhang B, Huang S-M, Iwata N, Saido TC, Maeda J, Suhara T, Trojanowski JQ, Lee VM-Y (2007). Synapse loss and microglial activation precede tangles in a P301S tauopathy mouse model. Neuron.

[50] Castillo-Carranza DL, Sengupta U, Guerrero-Muñoz MJ, Lasagna-Reeves CA, Gerson JE, Singh G, Estes DM, Barrett AD, Dineley KT, Jackson GR (2014). Passive immunization with tau oligomer monoclonal antibody reverses tauopathy phenotypes without affecting hyperphosphorylated neurofibrillary tangles. J Neurosci.

[51] Kaniyappan S, Chandupatla RR, Mandelkow E-M, Mandelkow E (2017). Extracellular low-n oligomers of tau cause selective synaptotoxicity without affecting cell viability. Alzheimers Dement.

[52] Santa-María I, Pérez M, Hernández F, Avila J, Moreno FJ (2006). Characteristics of the binding of thioflavin S to tau paired helical filaments. J Alzheimers Dis.

[53] Xue C, Lin TY, Chang D, Guo Z (2017). Thioflavin T as an amyloid dye: fibril quantification, optimal concentration and effect on aggregation. R Soc Open Sci.

[54] Flach K, Hilbrich I, Schiffmann A, Gärtner U, Krüger M, Leonhardt M, Waschipky H, Wick L, Arendt T, Holzer M (2012). Tau oligomers impair artificial membrane integrity and cellular viability. J Biol Chem.

[55] Karikari TK, Nagel DA, Grainger A, Clarke-Bland C, Hill EJ, Moffat KG (2019). Preparation of stable tau oligomers for cellular and biochemical studies. Anal Biochem.

[56] Graeber MB, Li W, Rodriguez ML (2011). Role of microglia in CNS inflammation. FEBS Lett.

[57] Wegmann S, Nicholls S, Takeda S, Fan Z, Hyman BT (2016). Formation, release, and internalization of stable tau oligomers in cells. J Neurochem.

[58] Wang C, Telpoukhovskaia MA, Bahr BA, Chen X, Gan L (2018). Endo-lysosomal dysfunction: a converging mechanism in neurodegenerative diseases. Curr Opin Neurobiol.

[59] Evans LD, Wassmer T, Fraser G, Smith J, Perkinton M, Billinton A, Livesey FJ (2018). Extracellular monomeric and aggregated tau efficiently enter human neurons through overlapping but distinct pathways. Cell Rep.

[60] Rauch JN, Chen JJ, Sorum AW, Miller GM, Sharf T, See SK, Hsieh-Wilson LC, Kampmann M, Kosik KS (2018). Tau internalization is regulated by 6-O sulfation on heparan sulfate proteoglycans (HSPGs). Sci Rep.

[61] Colton CA, Wilcock DM: Assessing activation states in microglia. CNS Neurol Disord-Drug Targets (Formerly Current Drug Targets-CNS & Neurological Disorders) 2010, 9:174–191. 10.2174/187152710791012053 20205642

[62] Meng X-L, Chen C-L, Liu Y-Y, Su S-J, Gou J-M, Huan F-N, Wang D, Liu H-S, Ben S-B, Lu J (2019). Selenoprotein SELENOK enhances the migration and phagocytosis of microglial cells by increasing the cytosolic free Ca2+ level resulted from the up-regulation of IP3R. Neuroscience.

[63] Wang L, Jiang Q, Chu J, Lin L, Li X-G, Chai G-S, Wang Q, Wang J-Z, Tian Q (2013). Expression of Tau40 induces activation of cultured rat microglial cells. PLoS One.

[64] Tsuda M, Masuda T, Kitano J, Shimoyama H, Tozaki-Saitoh H, Inoue K (2009). IFN-γ receptor signaling mediates spinal microglia activation driving neuropathic pain. Proc Natl Acad Sci.

[65] Uhlemann R, Gertz K, Boehmerle W, Schwarz T, Nolte C, Freyer D, Kettenmann H, Endres M, Kronenberg G (2016). Actin dynamics shape microglia effector functions. Brain Struct Funct.

[66] Elmore MRP, Hohsfield LA, Kramár E, Soreq L, Lee RJ, Pham ST, Najafi AR, Spangenberg EE, Wood MA, West BL (2018). Replacement of microglia in the aged brain reverses cognitive, synaptic, and neuronal deficits in mice. Aging Cell.

[67] Füger P, Hefendehl JK, Veeraraghavalu K, Wendeln A-C, Schlosser C, Obermüller U, Wegenast-Braun BM, Neher JJ, Martus P, Kohsaka S (2017). Microglia turnover with aging and in an Alzheimer’s model via long-term in vivo single-cell imaging. Nat Neurosci.

[68] Neiva I, Malva JO, Valero J (2014). Can we talk about microglia without neurons? A discussion of microglial cell autonomous properties in culture. Front Cell Neurosci.

[69] Liu H-C, Zheng M-H, Du Y-L, Wang L, Kuang F, Qin H-Y, Zhang B-F, Han H (2012). N9 microglial cells polarized by LPS and IL4 show differential responses to secondary environmental stimuli. Cell Immunol.

[70] Gorantla NV, Chinnathambi S (2018). Tau protein squired by molecular chaperones during Alzheimer’s disease. J Mol Neurosci.

[71] Sabbagh JJ, Dickey CA (2016). The metamorphic nature of the tau protein: dynamic flexibility comes at a cost. Front Neurosci.

[72] Gorantla NV, Shkumatov AV, Chinnathambi S. Conformational dynamics of intracellular tau protein revealed by CD and SAXS. In Tau Protein Springer. 2017;1523:3–20. 10.1007/978-1-4939-6598-4_1 27975241

[73] Mizuno Tetsuya (2012). The Biphasic Role of Microglia in Alzheimer's Disease. International Journal of Alzheimer's Disease.

[74] Kronenberg J, Merkel L, Heckers S, Gudi V, Schwab HM, Stangel M (2019). Investigation of neuregulin-1 and glial cell-derived neurotrophic factor in rodent astrocytes and microglia. J Mol Neurosci.

[75] Yates D (2018). Neurodegenerative disease: a proteostatic boost. Nat Rev Neurosci.

[76] Brelstaff J, Tolkovsky AM, Ghetti B, Goedert M, Spillantini MG (2018). Living neurons with tau filaments aberrantly expose phosphatidylserine and are phagocytosed by microglia. Cell reports.

[77] Hopp SC, Lin Y, Oakley D, Roe AD, DeVos SL, Hanlon D, Hyman BT (2018). The role of microglia in processing and spreading of bioactive tau seeds in Alzheimer’s disease. J Neuroinflammation.

[78] Vogels T, Murgoci A-N, Hromádka T (2019). Intersection of pathological tau and microglia at the synapse. Acta neuropathologica communications.

[79] Sogn CJ, Puchades M, Gundersen V (2013). Rare contacts between synapses and microglial processes containing high levels of Iba1 and actin–a postembedding immunogold study in the healthy rat brain. Eur J Neurosci.

[80] Rajan WD, Wojtas B, Gielniewski B, Gieryng A, Zawadzka M, Kaminska B (2019). Dissecting functional phenotypes of microglia and macrophages in the rat brain after transient cerebral ischemia. Glia.

[81] Franco-Bocanegra DK, McAuley C, Nicoll JA, Boche D (2019). Molecular mechanisms of microglial motility: changes in ageing and Alzheimer’s disease. Cells.

[82] Bollmann L, Koser DE, Shahapure R, Gautier HOB, Holzapfel GA, Scarcelli G, Gather MC, Ulbricht E, Franze K (2015). Microglia mechanics: immune activation alters traction forces and durotaxis. Front Cell Neurosci.

[83] Barcia C, Ros CM, Annese V, Sauvage C-d, Angeles M, Ros-Bernal F, Gómez a, Yuste JE, Campuzano CM, De Pablos V, Fernandez-Villalba E (2012). ROCK/Cdc42-mediated microglial motility and gliapse formation lead to phagocytosis of degenerating dopaminergic neurons in vivo. Sci Rep.

[84] Stuart LM, Bell SA, Stewart CR, Silver JM, Richard J, Goss JL, Tseng AA, Zhang A, El Khoury JB, Moore KJ (2007). CD36 signals to the actin cytoskeleton and regulates microglial migration via a p130Cas complex. J Biol Chem.

[85] Siddiqui TA, Lively S, Vincent C, Schlichter LC (2012). Regulation of podosome formation, microglial migration and invasion by Ca 2+ signaling molecules expressed in podosomes. J Neuroinflammation.

[86] Ohsawa K, Imai Y, Sasaki Y, Kohsaka S (2004). Microglia/macrophage-specific protein Iba1 binds to fimbrin and enhances its actin-bundling activity. J Neurochem.

