# Supplementary material for: Phagocytosis of full-length Tau oligomers by Actin-remodeling of activated microglia
Source: J Neuroinflammation. 2020 Jan 8;17:10. doi: 10.1186/s12974-019-1694-y (PMC6950897; doi:10.1186/s12974-019-1694-y)
Supplement: Supplementary file 1 — Additional file 1: Figure S1. TEM analysis of hTau40WTspecies and control groups. (A) hTau40WT oligomer forms globular structure as observed by TEM. (B) Soluble Tau fixed with glutaraldehyde were negatively stained with uranyl acetate, which formed no specified structures in TEM study. (C) The carbon mess grids stained with only uranyl acetate as negative control. (D) The SDS-PAGE analysis of hTau40WT fibrillar aggregate and soluble monomer [file 12974_2019_1694_MOESM1_ESM.pdf]

Supplementary Information

Phagocytosis of full-length Tau oligomers by Actin-remodeling of activated microglia

Rashmi Das[1, 2], Abhishek Ankur Balmik[1, 2], Subashchandrabose Chinnathambi[1, 2*]

[1]Neurobiology Group, Division of Biochemical Sciences, CSIR-National Chemical Laboratory, Dr. Homi Bhabha Road, 411008 Pune, India

[2]Academy of Scientific and Innovative Research (AcSIR), 411008 Pune, India

[*]To whom correspondence should be addressed**: Prof. Subashchandrabose Chinnathambi**, Neurobiology group, Division of Biochemical Sciences, CSIR-National Chemical Laboratory (CSIR-NCL), Dr. Homi Bhabha Road, 411008 Pune, India, Telephone: +91-20-25902232, Fax. +91-20-25902648. Email: s.chinnathambi@ncl.res.in

Supplementary Figure 1

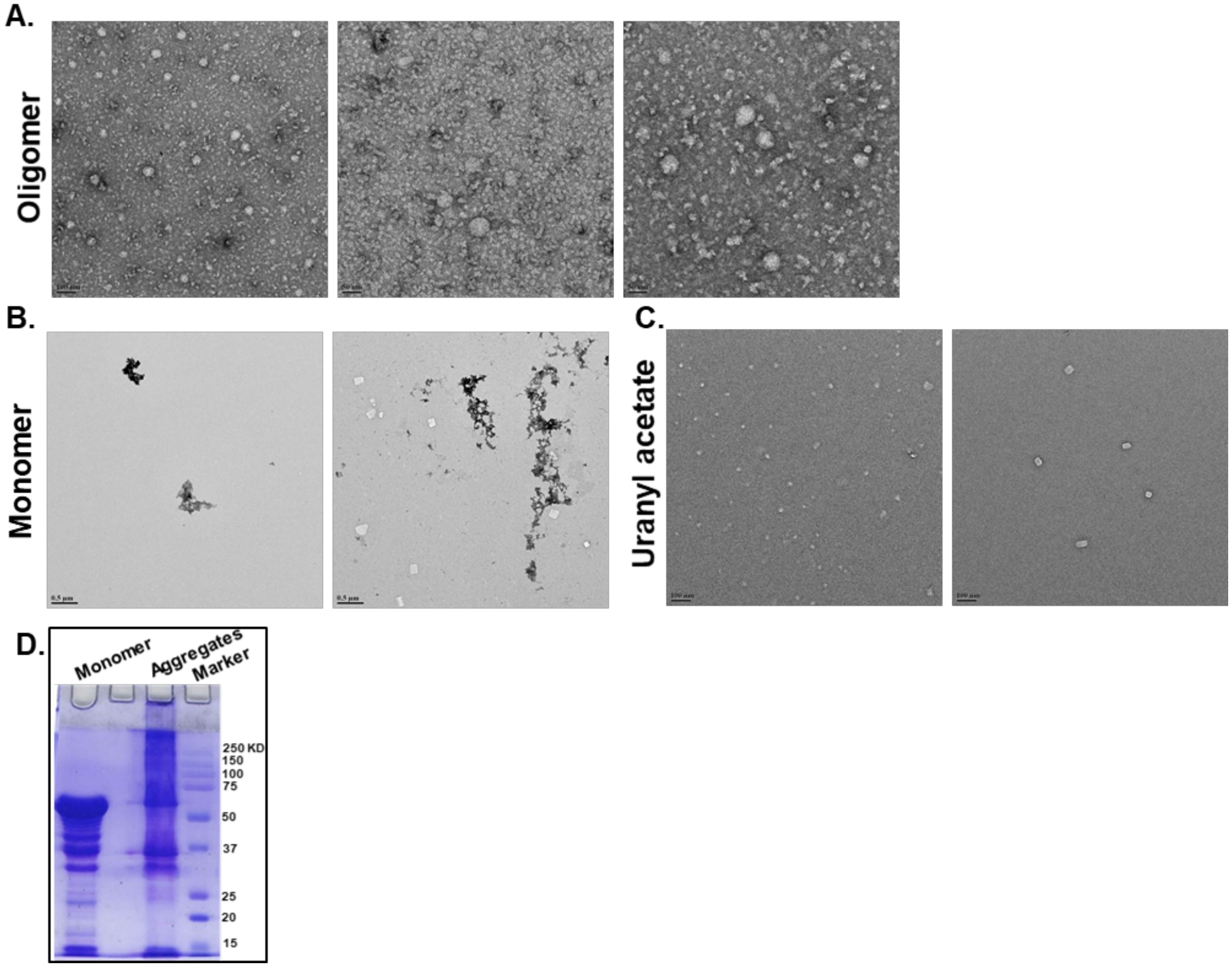

Supplementary figure 1. TEM analysis of HTau40^{WT} species and control groups. (A) hTau40^{WT} oligomer forms globular structure as observed by TEM. (B) Soluble Tau fixed with glutaraldehyde were negatively stained with uranyl acetate, which formed no specified structures in TEM study. (C) The carbon mess grids stained with only uranyl acetate as negative control. (D) The SDS-PAGE analysis of hTau40^{WT} fibrillar aggregate and soluble monomer.
